# Adrenocorticotropic Hormone-Secreting Pituitary Microadenoma Presenting with Acute Psychosis, Delirium and Paroxysmal Sympathetic Hyperactivity

**DOI:** 10.18295/squmj.6.2024.033

**Published:** 2024-08-29

**Authors:** Neeraja Rajeev, Abdullah M. Al-Fataisi, Rajeev Kariyattil

**Affiliations:** 1 Department of Internal Medicine, Sultan Qaboos University, Muscat, Oman; 2 Department of Surgery, Sultan Qaboos University, Muscat, Oman

**Keywords:** Cushing’s Disease, Neuropsychiatry, Dysautonomia, Case Report, Oman

## Abstract

Adrenocorticotropic hormone (ACTH)-secreting pituitary adenomas are known to be associated with behavioural changes but acute presentation including psychosis and delirium are less common. We report the case of a 42-year-old female patient with a known medical history of hypertension and diabetes mellitus, presenting with acute onset behavioural changes suggestive of psychosis to a tertiary care centre in Muscat, Oman in 2022. Further evaluation revealed an ACTH dependent Cushing’s disease with a pituitary microadenoma. The patient was admitted for endoscopic resection of the adenoma. During the peri-operative period, she experienced worsening of psychosis in addition to delirium. She also developed episodes of unresponsiveness, posturing, severe diaphoresis and dyspnoea accompanied by tachycardia and hypertension which were managed with midazolam and levetiracetam. A seizure work-up and computed tomography brain scan were unremarkable. At follow-up, she showed full resolution of symptoms with good blood pressure and glycaemic control.

Cushing’s disease (CD), caused by an adrenocorticotropic hormone (ACTH)-secreting pituitary adenoma is one of the commonest causes for Cushing’s syndrome (CS).^1^ Though neuropsychiatric manifestations are seen in approximately 70% of patients with CS, only 8% present with psychosis and agitation.^2^^,^^3^

## Case Report

**Figure 1:**
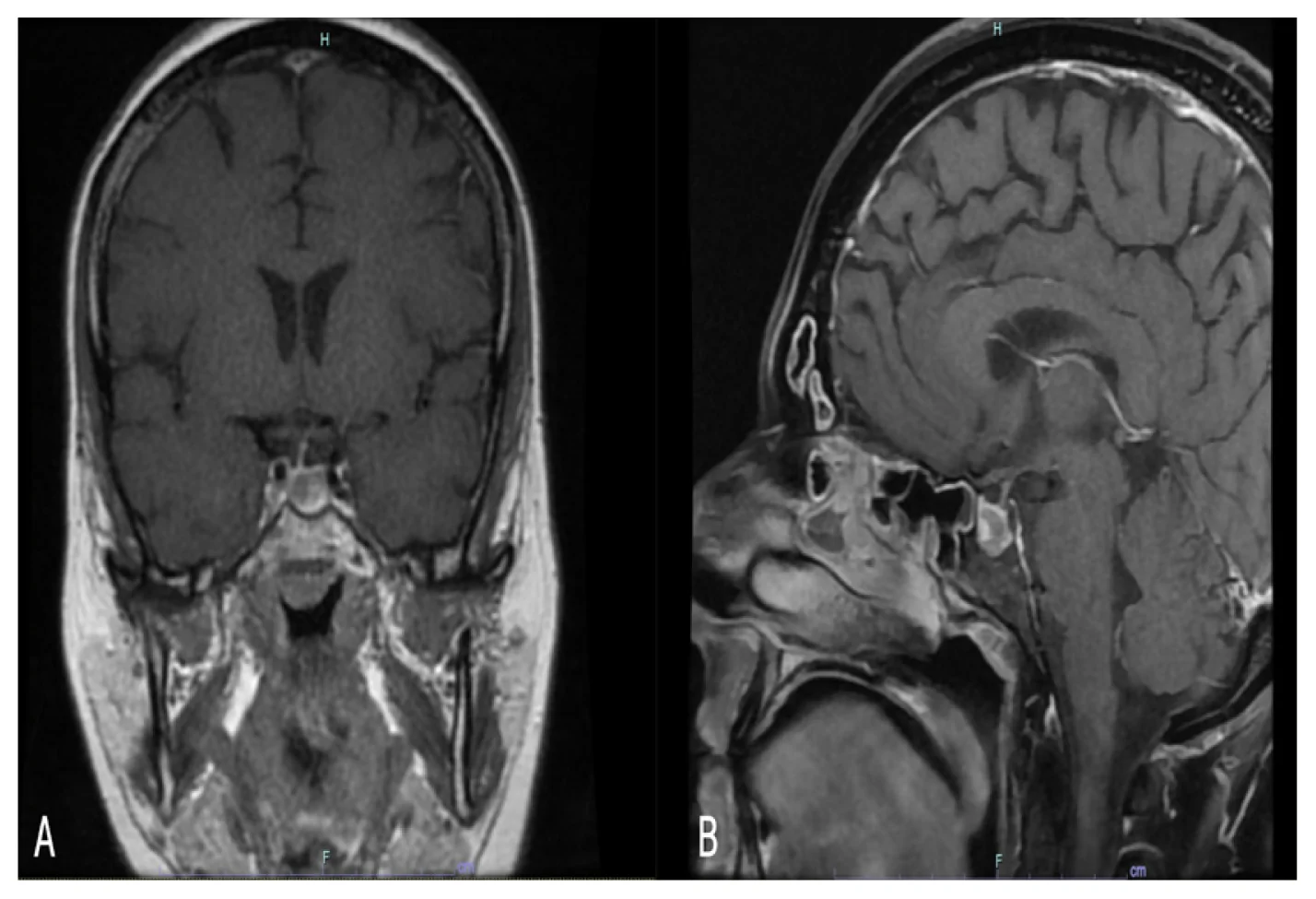
Pre-operative T1W magnetic resonance imaging scan with contrast in the (**A**) coronal and (**B**) sagittal view showing haemorrhagic pituitary microadenoma.

A 42-year-old female patient with a known medical history of diabetes, hypertension and hypothyroidism presented to another medical facility in 2022 with acute onset behavioural changes including irrelevant talk and agitation, suggestive of psychosis, which responded partially to anti-psychotic medication. She had no past history of behavioural changes. Further questioning revealed a history of recent weight gain and menstrual irregularity. Clinical examination revealed abdominal striae and cervico-thoracic fat deposition. Hormonal evaluation revealed serum cortisol level of 1,159 nmol/L (reference range: 133–537 nmol/L) and ACTH level of 97.9 ng/L (reference range: 7.2–63.3 ng/L) and a 24-hour urinary cortisol level of 3,063 nmol/L (reference range: 58–807 nmol/L). A magnetic resonance imaging (MRI) scan of the brain revealed a cup shaped hyperintensity on T1WI and T2WI images along the base of the sella. The left half of the sella was occupied by a hypointense non-enhancing nodule with a thin normally enhancing rim of pituitary gland seen on the right side. There was no suprasellar extension of this pituitary nodule. The findings were suggestive of a pituitary microadenoma with haemorrhagic changes [Figure 1].

She was referred to the authors’ institution, a tertiary care centre in Muscat, Oman, for further management. An endocrine evaluation with overnight dexamethasone suppression test showed a basal midnight cortisol level of 1,078 nmol/L and 833 nmol/L after 1 mg of dexamethasone, confirming a ACTH-dependent CD. All laboratory investigations to exclude other metabolic causes of delirium were within normal ranges. She was evaluated by the psychiatry team following worsening of her symptoms and a diagnosis of psychosis with delirium secondary to CD was made. She was managed with the anti-psychotic quetiapine along with intermittent haloperidol. Subsequently, she developed episodes of unresponsiveness, posturing, severe diaphoresis and dyspnoea accompanied by tachycardia (90–105 bpm), hypertension (200/110 mmHg) and oxygen desaturation which was treated with midazolam and levetiracetam for suspected seizures. The possibility of a diagnosis of paroxysmal sympathetic hyperactivity (PSH) was not considered at this time.

The following day, she underwent an endoscopic trans-nasal transsphenoidal excision of the adenoma. In the immediate post-operative period, she suffered from episodes of tachypnoea, hypertension and a decrease in oxygen saturation; this prompted a computed tomography (CT) pulmonary angiography for pulmonary embolism which was negative. CT brain and seizure evaluation were carried out which were also unremarkable. A serum creatinine kinase level was done to exclude neuroleptic malignant syndrome and was within normal limits. By the third day, the paroxysmal episodes subsided and she had become calm and coherent. She was discharged on the fifth post-operative day on quetiapine and levetiracetam. Her post-operative serum cortisol level was 121 nmol/L in the immediate post-operative period and 236 nmol/L after 1 month.

**Figure 2:**
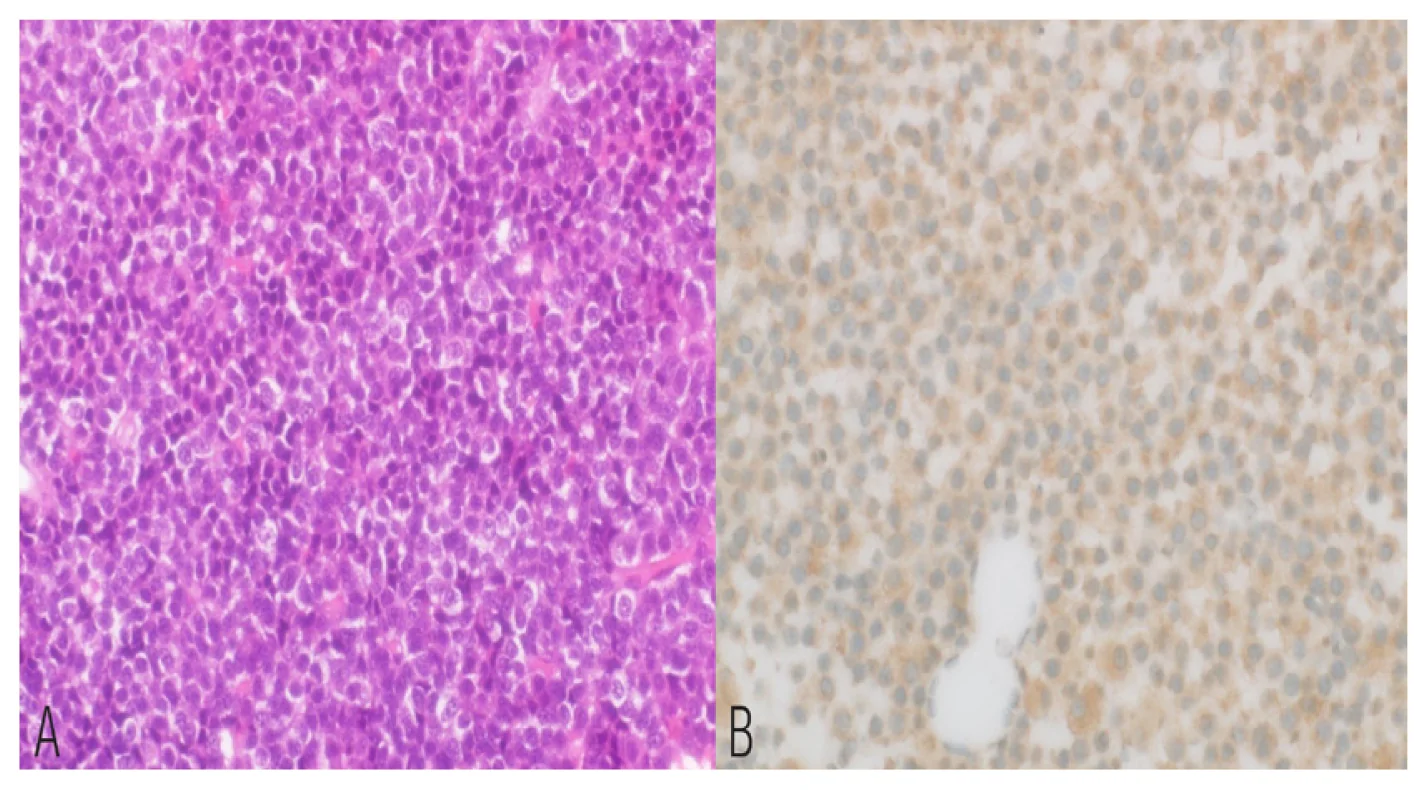
Tumour tissue consisting of groups and trabeculae of uniform cells with nuclear stippled chromatin, inconspicuous nucleoli and moderately abundant acidophilic cytoplasm using (**A**) haematoxylin and eosin stain at ×200 magnification; (**B**) the cells also show diffuse weak to moderate staining for adrenocorticotropic hormone using immunohistochemistry at ×200 magnification.

**Figure 3:**
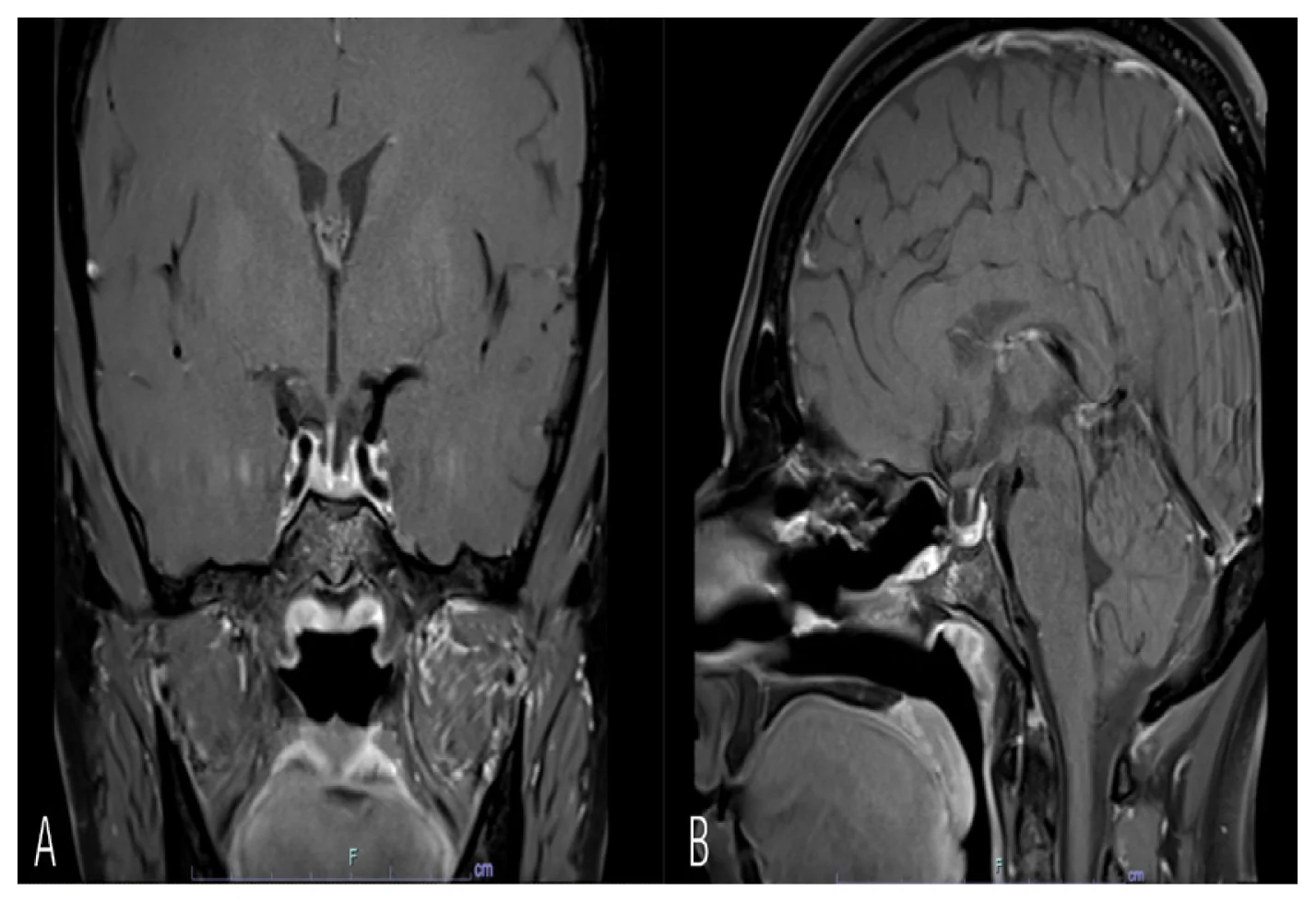
Post-operative T1W magnetic resonance imaging scan with contrast in the (**A**) coronal and (**B**) sagittal view showing no obvious residual.

By 2-weeks she had recovered significantly except for occasional involuntary facial twitching. An electroencephalogram done at follow-up was normal and therefore the anti-epileptic medication was discontinued. By 1 month she was asymptomatic and by 3 months she was off anti-psychotic medication. At 6 months follow-up, she had returned to her normal employment with good glycaemic and blood pressure control. The histopathology report revealed a corticotropin variant of pituitary adenoma [Figure 2]. An MRI scan of the pituitary on follow-up showed no obvious residual lesion [Figure 3].

This patient gave informed consent for publication purposes.

## Discussion

Neuropsychiatric manifestations are seen in approximately 70% of patients with CS at presentation and may be the presenting feature in 12% of patients.^2^ The most common symptom reported is depression which could not only be due to the hypercortisolism but also secondary to the other metabolic, skin and musculoskeletal effects and body image issues.^2^ Less commonly, approximately 8% present with psychosis and agitation.^2^^,^^3^ In the current case, the patient initially presented with sudden onset psychosis which progressed to delirium over a period of 1 week. The emergence of frequent episodes of hypertension, tachycardia and tachypnoea along with unresponsiveness and dystonic posturing was extremely unusual and prompted a differential diagnosis including seizures, pulmonary embolism and drug-induced neuroleptic malignant syndrome. However, when reviewed retrospectively, these episodes were very similar to the PSH episodes seen in severe head injury.^4^^,^^5^ When the PSH diagnostic assessment tool was applied to the current patient, a score of 18 was obtained which indicated a high probability of PSH.^5^

Though associated with severe head trauma in 80% of patients, PSH is rarely known to occur secondary to tumours especially with hypothalamic involvement.^6^ However, the current patient had a microadenoma with no supra-sellar extension and the post-operative imaging did not reveal any insult to the supra-sellar region. Investigations into the pathophysiology of PSH have shown a significant surge in levels of ACTH, epinephrine, norepinephrine and dopamine during the paroxysms.^4^ The current patient’s symptoms are also similar to the type A phaeochromocytoma crisis secondary to catecholamine release.^7^ In animal and human studies, ACTH has indeed been shown to influence adrenal medullary secretion either directly or indirectly through glucocorticoid stimulation.^8^–^10^ It could therefore, be hypothesised that the elevated ACTH levels resulted in a catecholamine surge which led to these PSH-like episodes preceding and immediately following surgery, with rapid resolution thereafter. Confirmatory tests such as plasma catecholamines and urinary metanephrine and normetanephrine could not be done as the diagnosis of PSH in this setting was never under consideration because it had not been previously reported.

Though medical management of neuropsychiatric symptoms (especially with cortisol lowering agents such as metyrapone and receptor blockers such as mifepristone) have been successfully used as an adjunct, definitive management is surgery with very good response, especially for patients with psychosis.^3^ Adjunctive management of the PSH-like symptoms include opiates, benzodiazepines, β-antagonists and bromocriptine.^4^ While it is difficult to ascertain, it seems plausible that the pre-operative medical management with cortisol lowering or receptor blocking agents reduced the severity of psychosis, delirium and possibly even the PSH-like symptoms in the current patient.

Though surgery provides a resolution of acute neuropsychiatric manifestations, more chronic symptoms such as depression may persist in many patients suggesting the need for long term follow-up.^2^

## Conclusion

The diagnostic work-up of new onset behavioural disturbance especially psychosis should involve a good metabolic and hormonal screening. Though medical management can reduce the symptoms of psychosis in CD, early surgery offers rapid and lasting resolution of symptoms. The features suggestive of sympathetic hyperactivity is an extremely unusual presentation for CD, requiring exclusion of other serious causes before attributing an association.
